# Fate of *Salmonella* Typhimurium and *Listeria monocytogenes* on Whole Papaya during Storage and Antimicrobial Efficiency of Aqueous Chlorine Dioxide Generated with HCl, Malic Acid or Lactic Acid on Whole Papaya

**DOI:** 10.3390/foods10081871

**Published:** 2021-08-12

**Authors:** Lianger Dong, Yong Li

**Affiliations:** Department of Human Nutrition, Food and Animal Sciences, University of Hawaii, 1955 East-West Road, Honolulu, HI 96822, USA; ldong@hawaii.edu

**Keywords:** whole papaya, *Salmonella* Typhimurium, *Listeria monocytogenes*, survival, aqueous chlorine dioxide, malic acid, shelf-life

## Abstract

Papaya-associated foodborne illness outbreaks have been frequently reported worldwide. The goal of this study was to evaluate the behavior of *Salmonella* Typhimurium and *Listeria monocytogenes* on whole papaya during storage and sanitizing process. Fresh green papayas were inoculated with approximately 7 log CFU of *S.* Typhimurium and *L. monocytogenes* and stored at 21 or 7 °C for 14 days. Bacteria counts were determined on day 0, 1, 7, 10 and 14. Fresh green papayas inoculated with approximately 8 log CFU of the bacteria were treated for 5 min with 2.5, 5 and 10 ppm aqueous chlorine dioxide (ClO_2_). The ClO_2_ solutions were generated by mixing sodium chlorite with an acid, which was HCl, lactic acid or malic acid. The detection limit of the enumeration method was 2.40 log CFU per papaya. At the end of storage period, *S.* Typhimurium and *L. monocytogenes* grew by 1.88 and 1.24 log CFU on papayas at 21 °C, respectively. Both bacteria maintained their initial population at inoculation on papayas stored at 7 °C. Higher concentrations of ClO_2_ reduced more bacteria on papaya. 10 ppm ClO_2,_ regardless the acid used to generate the solutions, inactivated *S.* Typhimurium to undetectable level on papaya. 10 ppm ClO_2_ generated with HCl, lactic acid and malic acid reduced *L. monocytogenes* by 4.40, 6.54 and 8.04 log CFU on papaya, respectively. Overall, ClO_2_ generated with malic acid showed significantly higher bacterial reduction than ClO_2_ generated with HCl or lactic acid. These results indicate there is a risk of survival and growth for *S.* Typhimurium and *L. monocytogenes* on papaya at commercial storage conditions. Aqueous ClO_2_ generated with malic acid shows effectiveness in inactivating the pathogenic bacteria on papaya.

## 1. Introduction

Papaya (*Carica papaya*) is one of the major tropical agricultural commodities amongst banana, mango, avocado and pineapple [1]. Annual global papaya production has increased by approximately 90% since 2000 and reached 13.7 million metric tons in 2019 [2]. The top three papaya-producing countries are India, Brazil and Mexico, among which 99% of Mexican papayas are exported to the United States [2]. However, along with the increased papaya demand and production worldwide, foodborne illness outbreaks linked to papaya have also been emerging in recent years [3,4]. In particular, outbreaks associated with whole fresh papaya have been frequently reported in the U.S. from 2011 to 2019, which affected the papaya industry in both US and Mexico [4,5]. Papaya grows best in tropic environments at 21–33 °C where the survival and growth of pathogenic bacteria are favored [6]. Microbial contamination of papaya might happen at any step of the production chain where the fruits are in contact with water, soil, harvest equipment and human handling [7]. *Salmonella* Litchfield was detected on whole papayas associated with an outbreak in Australia between 2006 and 2007, and other *Salmonella* serotypes of Chester, Eastborne and Poona were detected in farm water samples [3]. In multiple cases reported in the U.S., whole papayas were contaminated by *Salmonella* serotypes of Agona, Uganda, Newport, etc. [5]. Therefore, papaya seems to be susceptible to *Salmonella* contamination. In addition, *Listeria monocytogenes* is one of the concerned foodborne pathogenic bacteria associated with fresh produce due to its nature of being present in the environment and its ability to grow at refrigeration temperature [8]. *L. monocytogenes*-caused multistate outbreaks in the U.S. were linked to whole cantaloupe and caramel apple [9,10]. *L. monocytogenes* was also found to be able to survive or grow on the surfaces of apple, mango, kiwifruit and cherry tomato under various storage conditions [11,12,13,14].

Studies have reported the survival and growth of foodborne pathogenic bacteria in fresh-cut papaya and papaya pulp [15,16,17,18,19]. However, little is known regarding whole fresh papaya. There are differences between fresh-cut and whole fruits in terms of pH, nutrient availability and native microflora composition. For example, *S.* Typhimurium and *L. monocytogenes* decreased by approximately 2–2.5 log CFU over 20 days on whole mango at 25 °C; however, these bacteria grew on cut mango [12]. The growth of *L. monocytogenes* was inhibited on intact jalapeño pepper stored at 7 °C for 14 days, but it grew in the internal cavity of jalapeño pepper at the same storage condition [20]. It is important to note that even when the skin part of fruit is inedible or usually not eaten, pathogenic bacteria surviving on the surface may further cross-contaminate wash water and other fruits that are rinsed in the same batch, internalize into the flesh or transfer to fruit flesh during cutting [21,22]. Information of pathogenic bacteria behavior on whole papaya would assist regulatory and industrial agencies in the assessment and prevention of papaya microbiological safety issues.

Once contaminated, fresh fruits cannot be thermally disinfected and would likely be distributed to the market. Therefore, washing and sanitizing is a critical step in the post-harvest process to prevent cross-contamination and reduce pathogens. Chlorine-based bleach at a concentration of 50–200 ppm is the most widely used sanitizer in fresh produce handling and processing [23]. However, the effectiveness of chlorine varies at different pH and is reduced significantly in the presence of organics, and there are concerns regarding the carcinogenetic by-products such as trihalomethanes formed in the reactions between chlorine and organics [24]. Chlorine dioxide (ClO_2_) is approved by FDA for fresh produce washing with a maximum residue of 3 ppm in the wash water [24]. The antimicrobial efficacy of ClO_2_ is less prone to low pH and the presence of organics than chlorine [25]. ClO_2_ also forms fewer carcinogenetic by-products than chlorine when chlorinated [24]. Despite the advantages, ClO_2_ is reduced to chlorite (ClO^2−^), chlorate (ClO^3−^) and chloride (Cl^−^) to some extend [26]. The United State Environmental Protection Agency (EPA) sets the Maximum Residual Disinfectant Level (MRDL) of ClO_2_ in public drinking water to be 0.8 mg/L and the Maximum Contaminant Level (MCL) of ClO^2−^ to be 1.0 mg/L [27]. ClO_2_ has been studied in sanitizing a wide variety of fresh produce, such as lettuce, cantaloupe, alfalfa sprouts and blueberries [23,28,29,30]. No ClO_2_, ClO^2−^ or ClO^3−^ residues were detected in Mulberry fruit treated by 60 ppm aqueous ClO_2_ for 15 min [31]. Cantaloupes, oranges, tomatoes and apples treated with 5 ppm gaseous ClO_2_ for 10 min showed very minimal ClO^2−^ residue on the fruits with a maximum of 0.36 mg/kg; however, lettuce and alfalfa sprouts had high ClO^2−^ residue of 16.5–1259.6 mg/kg [32]. Acidified sodium chlorite was used to reduce microbial contamination in shredded green papaya [33]. Ozone was used to reduce the microbial load and improve the nutritional values of fresh-cut papaya [34]. Gu et al. investigated the efficiency of chlorine or peracetic acid in the inactivation and cross-contamination prevention of *Salmonella* spp. on Maradol papayas [35]. Inactivation of pathogenic bacteria by ClO_2_ has not been investigated on whole papayas.

Aqueous ClO_2_ can be made by mixing an acid with sodium chlorite (NaClO_2_) [36]. Hydrochloric acid (HCl) is a commonly used acid in ClO_2_ generation [30,31,32,36]. Kim et al. [37] reported ClO_2_ solutions formed from organic acids, including acetic acid, citric acid and lactic acid, were more stable and more lethal to *Bacillus cereus* spores than ClO_2_ formed using HCl. Our previous study has also shown that aqueous ClO_2_ generated by mixing NaClO_2_ with organic acids, including citric acid, lactic acid and malic acid, had higher antimicrobial efficacy against common foodborne pathogenic bacteria on Romaine lettuce than ClO_2_ generated with inorganic acids [38]. For example, 5 min treatments with 5 ppm ClO_2_ generated with lactic acid, citric acid and malic acid reduced S. Typhimurium on Romaine lettuce by 0.92, 1.39 and 1.37 log CFU/g, respectively, whereas lettuce treated with ClO_2_ generated with HCl and sodium bisulfate reduced *S*. Typhimurium by 0.71 and 1.14 log CFU/g, respectively [38].

In numerous studies investigating the survival of foodborne pathogenic bacteria on fresh produce or decontamination of fresh produce using sanitizers, procedures used to recover and quantify bacteria cells from fresh produce vary. The ununiformed procedures make it difficult to compare and accurately interpret results of different studies [39]. For example, pummeling using a stomacher resulted in higher bacteria recovery than pulsifying, sonication and shaking by hand from iceberg lettuce, perilla leaves, cucumber and green pepper, while a lower level of bacteria was recovered from cherry tomato due to its acidity [40]. Sample preparation method, bacteria type and produce type may affect the efficiency of bacteria recovery and hence further affect the accuracy of a microbiological method. So far, there has been no recommendation of sample preparation methods specifically for whole papaya.

This study aimed to optimize homogenization parameters and enumeration methods for recovering *S.* Typhimurium and *L. monocytogenes* from papaya surface. It also sought to evaluate the behaviors of these pathogenic bacteria on whole papaya during storage and sanitizing process. Obtaining information in this regard would assist the papaya industry in selecting optimal sanitizer type, usage concentration and treatment time for papaya washing and sanitizing.

## 2. Materials and Methods

### 2.1. Bacterial Strains and Cell Cultures

*Salmonella* Typhimurium (ATCC 14028) and *Listeria monocytogenes* (F2365) were obtained from Food Microbiology Lab at the University of Hawaii at Manoa and stored in trypticase soy broth (TSB; Becton Dickinson, Franklin Lakes, NJ, USA) containing 50% glycerol at −80 °C. Working cultures were prepared by transferring 50 µL of stock culture into 5 mL of sterile TSB and incubating at 37 °C for 24 h. Working cultures were transferred twice in TSB before each experiment.

### 2.2. Preparation of Papayas and Inocula

Fresh papayas (*Carica papaya* L.cv. Rainbow Solo) were purchased on the day of experimentations on separate occasions from local grocery stores in Honolulu, USA. Non-injured whole papayas at mature green/color break stage were selected according to the maturity chart [41]. Papayas were rinsed with tap water and dried on a lab bench at room temperature for 1 h. Then an area of 2.5 × 2.5 cm^2^ on the middle part of the fruit surface was marked with a thin-line non-toxic marker (Sharpie, Oak Brook, IL, USA). The marked whole papayas were placed on sterile Petri dishes in a biosafety hood before experimenting. *S.* Typhimurium and *L. monocytogenes* cultures were diluted with 0.1% peptone water (Becton Dickinson, Franklin Lakes, NJ, USA) to desired concentrations. 100 µL of the inoculum was spot inoculated on the marked area and the papayas were dried under a biosafety hood. For Section 2.3 and Section 2.4, approximately 10^7^ log CFU of *S.* Typhimurium or *L. monocytogenes* inocula were used, and the papayas were dried for 1 h to initiate the attachment before every experiment [42]. For Section 2.5, approximately 10^8^ log CFU of the inocula were used, and the papayas were dried for two hours to ensure attachment and initiate colonization before being washed with sanitizer solutions [42].

### 2.3. Optimization of Recovery Method for Counting Bacteria Cells on Papaya Surface

#### 2.3.1. Recovery Method

Optimization of homogenization parameters is essential for accurate assessment of bacterial behavior on fruit surfaces. The goal of this experiment was to maximize the number of bacteria cells recovered from the papaya surface. After inoculation and drying as described above, the skin of the inoculated area was excised with a sterile knife and placed in a sterile stomacher bag. Bacterial cells were collected by homogenizing the skin under different conditions described as follows. Tested homogenization buffers included phosphate buffered saline (PBS, pH 7.4), 0.1% peptone water (PEPT), PBS + 0.2% Tween 80 (PBS + T) and 0.1% peptone water + 0.2% Tween 80 (PEPT + T). 25 mL of each buffer was separately added into the stomacher bag containing the excised skin and homogenized at 150 or 250 rpm for 1 or 5 min using a stomacher (Seward Stomacher^®^, Model 400 Circulator, West Sussex, UK). After homogenization, the homogenate was serially diluted with 0.1% peptone water and plated on selective agar or using the agar overlay method. The agar overlay method was to plate the serially diluted homogenate on Plate Count Agar (PCA, Becton Dickinson, Franklin Lakes, NJ, USA) and incubating the plate at 37 °C for 1 h to ensure the recovery of injured cells, followed by pouring warm selective agar at 55 °C over the PCA [43]. The agar plates were incubated at 37 °C for 24 h and then analyzed for bacterial counts. The selective agar for *S.* Typhimurium and *L. monocytogenes* were xylose lysine deoxycholate agar (XLD, Becton Dickinson, Franklin Lakes, NJ, USA) and modified oxford agar (MOX, Becton Dickinson, Franklin Lakes, NJ, USA), respectively. Bacterial colonies were counted and populations were expressed as log CFU/papaya. The detection limit was 2.40 log CFU/papaya.

#### 2.3.2. PH of Papaya Skin Homogenate as Affected by Homogenization Parameters

Papayas were prepared as described in Section 2.2 except that they were not inoculated with pathogenic bacteria. The skin of the marked area was cut and homogenized with buffer in a stomacher bag under the conditions described above. Papaya skin was also homogenized with water as a control. pH of the homogenate was measured using a pH meter (Model pH 6+, Oakton Instruments, Vernon Hills, IL, USA).

### 2.4. Behavior of Pathogenic Bacteria on Whole Papayas Stored at Different Temperatures

After harvesting and packing, papayas are usually stored at 7–13 °C before being distributed to grocery stores [44]. At grocery stores and customers’ homes, papayas are usually placed at room temperature (21–25 °C). Hence, we selected 21 and 7 °C to simulate the two papaya storage scenarios. Inoculated whole papayas were individually placed in large sterile beakers and stored at 21 and 7 °C for 14 days. One papaya was randomly sampled, with the skin of the inoculated area being sterilely excised and collected for bacteria count on storage days 0, 7, 10 and 14. The papaya that was inoculated and dried for 1 h on the day of inoculation was considered as the sample on day 0. To determine bacterial population on papaya, the excised skin was homogenized using the optimized method from Section 2.3, which was homogenizing in PBS + T buffer at 250 rpm for 1 min for both *S.* Typhimurium and *L. monocytogenes*. Subsequently, the homogenates were serially diluted with 0.1% peptone water and plated using the agar overlay method described above. After incubation, bacterial colonies were counted and populations were expressed as log CFU/papaya.

### 2.5. ClO_2_ Treatment on Whole Papayas

#### 2.5.1. Preparation of Aqueous ClO_2_

Aqueous ClO_2_ solutions were made on-site using a previous method [38]. Briefly, ClO_2_ stock solutions were prepared by mixing 10 mL of 4.0% NaClO_2_ (Fisher Scientific, Waltham, MA, USA) with 10 mL of 1 M HCl (Fisher Scientific, Waltham, MA, USA), lactic acid (VWR Chemicals, Radnor, PA, USA) or malic acid (Fisher Scientific) in aluminum foil-covered bottles. After reacting for 1 min, 100 mL of distilled water was added into the bottles. The final mixture was set at 21 °C for 20 min before being placed in a refrigerator at 4 °C. We previously investigated the generation kinetics and the stability of ClO_2_ [38]. As organic acids release hydrogen ions slowly, it took one week to achieve equilibrium. During the 14-day-experimentation, the ClO_2_ concentration increased till up to day seven and then remained stable for those generated with organic acids. For ClO_2_ generated with HCl, the reaction was quick and the concentration remained stable for up to eight days and eventually decreased. Therefore, the stock solutions were all stored for seven days to allow the completion of the reaction in malic acid- and lactic acid-produced ClO_2_ solutions and ensure no loss of the effectiveness of HCl-produced ClO_2_ solutions. On the day of experimentation, the concentration of ClO_2_ in each stock solution was measured using Chlordioxid-Test kit (EMD Millipore Corp., Burlington, MA, USA). The stock solutions were diluted with distilled water to 2.5, 5 and 10 ppm to treat papayas. The pH of each diluted solution was determined.

#### 2.5.2. Washing Papayas with Aqueous ClO_2_ and Individual Acid Solutions

To wash artificially contaminated papayas, each papaya was inoculated with *S.* Typhimurium or *L. monocytogenes* as described in Section 2.2 and then submerged into a sterile container containing 1 L of ClO_2_ made with HCl, lactic acid or malic acid at concentrations of 2.5, 5 and 10 ppm. The submerged papayas were mildly stirred at a rate of 150 rpm for 5 min [45]. Subsequently, the washed fruits were dried under a biosafety hood for 15 min. After drying, the marked surface was sterilely cut and homogenized in 25 mL of PBS + T buffer at 250 rpm for 1 min. The homogenate was serially diluted and plated by the agar overlay method with XLD and MOX agar for the selection of *S.* Typhimurium and *L. monocytogenes*, respectively. Bacterial populations were expressed as log CFU/papaya, and the detection limit was 2.40 log CFU/papaya. Washing with distilled water and 200 ppm bleach (sodium hypochlorite (NaClO), pH 6.5) diluted from Clorox^®^ (6.0% NaClO, The Clorox Company, Oakland, CA, USA) served as the control treatments.

Acid solutions were prepared by adjusting 1 L of distilled water individually with 1 M HCl, 1 M lactic acid or 1 M malic acid to the pH of 10 ppm ClO_2_ made with the corresponding acid. Papayas inoculated with *S.* Typhimurium or *L. monocytogenes* were washed with the acid solutions, and the remaining bacteria were collected and enumerated following the procedures described above.

#### 2.5.3. ClO_2_ Residue on Papaya Surface after Washing

Papayas were washed with tap water and dried on a lab bench for 1 h. Subsequently, the papayas were washed with 1 L of ClO_2_ made with HCl, lactic acid or malic acid at concentrations of 5, 10 and 20 ppm. After drying for 15 min, the papayas were placed in 1-gallon Ziploc bags containing 100 mL distilled water. The papayas surfaces were hand massaged and rinsed thoroughly for 2 min, followed by filtering the rinse water into a flask [46]. 10 mL of the filtrate was collected and measured for ClO_2_ concentration using Chlordioxid-Test kit. The detection limit was 0.02 mg/L in the undiluted filtrate. The ClO_2_ concentration was converted into mg/kg papaya.

### 2.6. Statistical Analysis

All experiments were conducted in three independent replicates. Bacterial cultures were separately grown following the same procedure for each replicate. ClO_2_ solutions were prepared freshly for each replicate. Data were reported as mean ± standard deviation (SD). Analysis of variance and Tukey’s multiple comparison test were performed using SSPS software (IBM^®^ SPSS^®^ Statistics 24.0 for Windows, IBM Corp., Armonk, NY, USA). A significance level of 0.05 was used to determine the differences between the means of treatment groups.

## 3. Results and Discussion

### 3.1. Recovery of S. Typhimurium and L. monocytogenes Cells from Whole Papaya Surface as Affected by Homogenization Parameters and Enumeration Methods

Statistical analysis revealed no interactions among homogenization parameters, and only buffer significantly affected the bacterial count (*p* < 0.05). For *S.* Typhimurium (Table 1), papayas homogenized in buffers with the non-ionic surfactant Tween 80 resulted in significantly higher bacteria counts than those homogenized in peptone water alone. Tween 80 interrupts the hydrophobic interactions between bacteria cells and papaya surface and promotes the detachment of cells [47]. Papayas homogenized in the combination of PBS and Tween 80 (PBS + T) had the highest *S.* Typhimurium counts; an average of 5.36 log CFU was recovered from the initial inoculum of approximately 7 log CFU. Among all treatments, homogenization at 150 rpm for 5 min using XLD plating resulted in the highest recovery of 5.64 log CFU from papaya surface. For *L. monocytogenes* (Table 2), homogenization in PBS + T collected significantly more cells than in PBS alone (*p* < 0.05). Homogenization time, speed or plating method did not play a significant role in the collection. Homogenization at 150 rpm for 5 min by the agar overlay method resulted in the highest count of 5.09 log CFU. However, homogenization at 250 rpm for 1 min also resulted in relatively high *L. monocytogenes* counts. Homogenization at 250 rpm for 1 min was chosen for collecting *S.* Typhimurium and *L. monocytogenes* from papaya surface to maintain the time efficiency and consistency of the experiment. Even though the agar overlay method did not result in significantly higher bacteria counts than using selective agar alone, incubating on non-selective media before adding selective media would help recover bacteria cells injured by sanitizers [43]. It is an essential step to avoid over-estimation of the antimicrobial efficiency of sanitizers. Therefore, homogenizing the inoculated papaya piece in PBS + T at 250 rpm for 1 min was chosen, and the homogenate was decided to be plated by overlaying selective agar on PCA.

pH values of the above-mentioned homogenates were measured with uninoculated samples to compare buffering capacity between homogenization buffers. Even with careful excision, papaya flesh attached to the skin could acidify the homogenate. Papaya flesh has a pH of 4.87–5.7 [16,18]. This pH range does not inhibit the growth of *S.* Typhimurium or *L. monocytogenes*; however, it could influence the recovery of cells injured by desiccation [43]. Tian et al. incubated sublethally injured *E. coli* O157:H7 cells in nutrient broth at pH 4.0, 5.0, 6.0, 7.2 and 8.0. They found that the cells showed no significant recovery at pH 4.0 and 8.0 whereas the cells recovered by 0.48, 0.49 and 0.72 log CFU/mL in pH 5.0, 6.0 and 7.2, respectively, indicating that pH even at relatively high levels (5.0 and 6.0) did affect the recovery of sublethally injured cells [48]. Shown in Table 3, homogenizing papaya skin in different buffers resulted in significant differences in homogenate acidity in a descent order of PBS, PBS + T, PEPT, water and PEPT + T (*p* < 0.05). The initial pH value of each buffer was measured with PBS, PBS + T and water being neutral whereas PEPT and PEPT + T being slightly acidic (pH = 6.5–6.7). PBS is known for its high buffering capacity, whereas water and peptone water have little buffering capacity. When mixed with the papaya juice, the pH of water and peptone water decreased to 5.89–6.26. The pH of the homogenate may affect the state of cells, and this is consistent with the higher cell counts observed in PBS + T. Peptone water is often used in studies involving fresh produce [20,23,49]. Researchers should carefully select homogenization buffers since peptone water alone may lead to experimental errors in studies with acidic produce.

### 3.2. Behavior of Pathogenic Bacteria on Whole Papayas Stored at Different Temperatures

**Figure 1:**
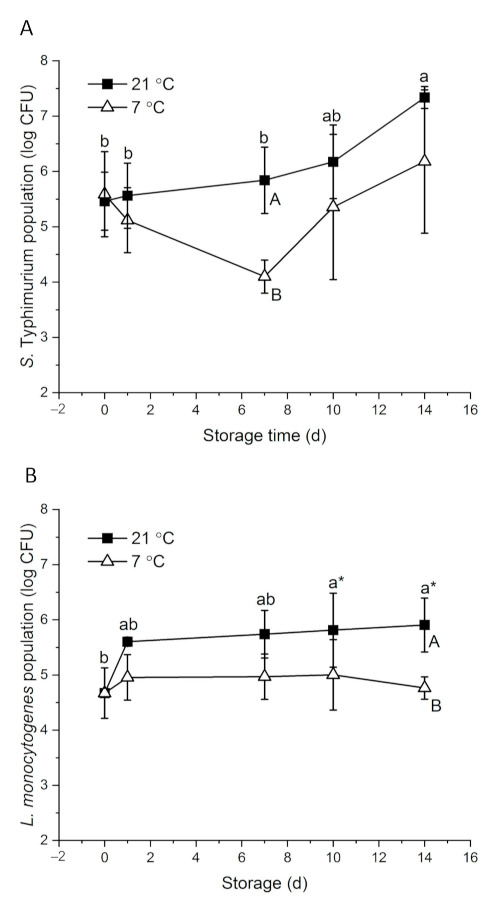
Behavior of *S.* Typhimurium (**A**) and *L. monocytogenes* (**B**) on whole papayas at 21 and 7 °C. Error bars are standard deviations (*n* = 3). Different lower-case letters horizontally indicate significant differences between the means of different time points at each temperature (*p* < 0.05). Different upper-case letters vertically indicate significant differences (*p* < 0.05) between the means of different temperatures at the same time point. “a*” means *p* values were marginal, which were 0.058 and 0.059 on day 10 and day 14, respectively, compared with day 0.

With about 7 log CFU of initial inocula, 5.46 and 4.67 log CFU *S.* Typhimurium and *L. monocytogenes* were detected on papaya surfaces on day 0, respectively (Figure 1). Bacteria response to environmental stress differently. *Salmonella* showed higher desiccation tolerance than *L. monocytogenes* in powdered infant formula and desiccated shredded coconut [50,51]. *S.* Typhimurium had an interesting survival and growth pattern. At 21 °C, the population increased gradually to 7.34 log CFU on day 14. At 7 °C, *S.* Typhimurium level decreased to 4.10 log CFU on day 7 and then increased to 6.18 log CFU at the end of the storage period (Figure 1A). Intrinsic factors of fruit, including surface roughness, surface hydrophobicity, nutrient and moisture availability and background flora, may affect the behavior of foodborne pathogenic bacteria on the fruit [8]. At ambient temperature, *S. enterica* level remained stable on whole mangos stored at 20–22 °C for nine days [52]. *Salmonella* was reduced by about 5 and 2 log CFU at high (~7 log) and low (~4 log) inoculation levels, respectively, on whole kiwifruits stored at room temperatures for 10 days [14]. On whole cucumbers stored at 23 °C, *Salmonella* level significantly increased by 1.7 log CFU within the first day of inoculation and remained stable for four days [53]. Looking at the fruit type alone, at commercial cold storage conditions (7–12 °C), *S.* Typhimurium level did not significantly change on whole papaya or mango at the end of the storage period [54]. However, *Salmonella* tended to decrease over time on other fruits, such as passionfruit, strawberry, cucumber and peppers [53,54,55,56]. Different from other tropical fruits, sugar accumulates on papaya surfaces as ripening progresses, which provides more nutrients for the attached microorganisms. Naturally present yeast may also aid the growth of *S.* Typhimurium by their saccharolytic interactions with the compounds permeated through papaya skin [57].

*L. monocytogenes* showed a major increase from 4.67 to 5.60 log CFU during the 1st day of storage at 21 °C, and then gradually grew to 5.91 log CFU in the following 13 days. At 7 °C, *L. monocytogenes* level remained stable on papayas for 14 days (Figure 1B). The behavior of *L. monocytogenes* on fruits varies. *L. monocytogenes* grew on whole cucumbers stored at 4 °C and grew on fresh Gala apples stored at 5 °C and 25 °C [53,57]. However, on Granny Smith apples, 1.5 log CFU and 0.5–1.2 log CFU reductions were observed at 25 and 22 °C, respectively, in two studies [13,57]. The reductions of *L. monocytogenes* on whole cantaloupe and mango were also reported [12,49]. Aside from the intrinsic differences of the fruits, initial inoculation levels and the carrying capacity of the fruit may contribute to the varied behavior of *L. monocytogenes* [8,18]. Approximately three-fold more *L. monocytogenes* died on whole kiwi fruits inoculated with 7 log CFU than those inoculated with 4 log CFU at room temperature over 10 days [14]. In the case of organic Granny Smith apples, *L. monocytogenes* decreased by 1.8 and 0.7 log CFU at inoculation levels of 6.3 and 3.0 log CFU, respectively, at 22 °C over two weeks [13]. Papayas could have a higher carrying capacity than the above-mentioned fruits, leading to the growth of *L. monocytogenes* on papayas even at a relatively high inoculation level. Regardless, *L. monocytogenes* is known for its ability to adapt to cold temperatures through mechanisms of alternating membrane fatty acid composition, synthesizing cold shock proteins and cold acclimation proteins and activating energy providing pathways such as glycolysis [58].

*S.* Typhimurium and *L. monocytogenes* showed abilities to survive and grow on papaya, and hence effective sanitation methods are essential for papaya production.

### 3.3. Inactivation of S. Typhimurium and L. monocytogenes on Whole Papayas Using Aqueous ClO_2_

**Figure 2:**
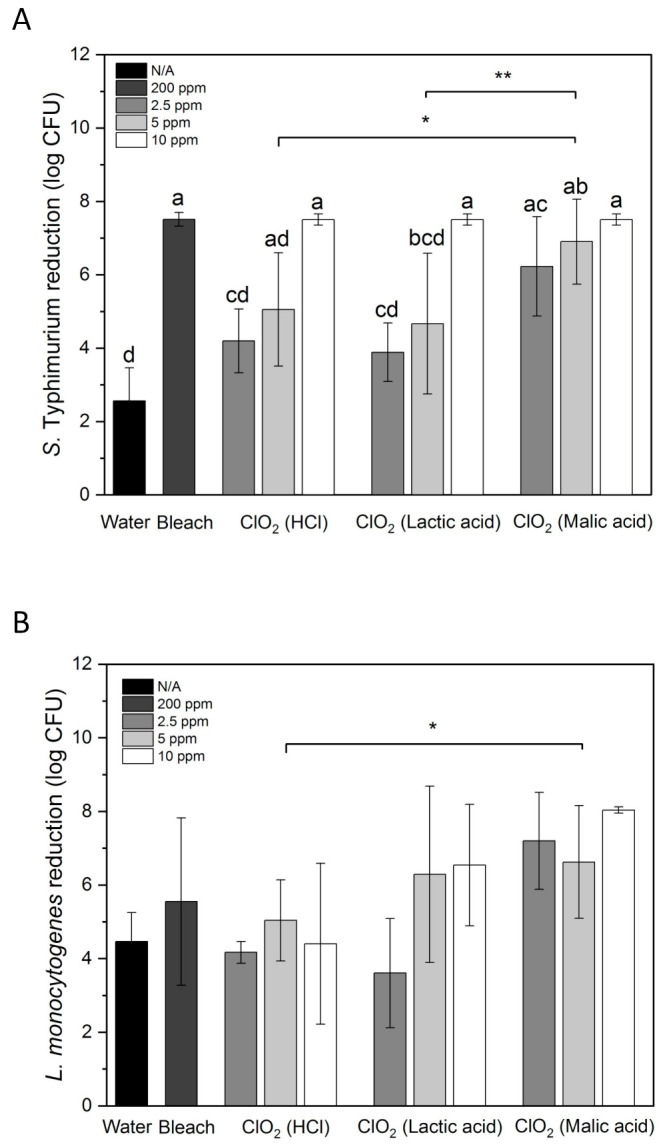
*S.* Typhimurium (**A**) and *L. monocytogenes* (**B**) reduction by water, 200 ppm bleach, and aqueous ClO_2_ generated by mixing NaClO_2_ with HCl, lactic acid or malic acid on whole papayas. Error bars are standard deviations (*n* = 3). Bars labeled with different letters indicate significant differences between the means of treatments (*p* < 0.05). Lines labeled with “*” indicate significant differences between ClO_2_ groups made with different acids (“*”, *p* < 0.05; “**”, *p* < 0.01).

Figure 2A shows *S*. Typhimurium reduction by water, aqueous ClO_2_, and bleach on whole papayas. 10 ppm of ClO_2_ was significantly more effective than 2.5 and 5 ppm (*p* < 0.05). 10 ppm of ClO_2_ reduced *S*. Typhimurium from the initial inoculation level of 7.5 log CFU to an undetectable level. 200 ppm of bleach achieved the same result. Malic acid-produced ClO_2_ reduced *S*. Typhimurium by 6.23 and 6.90 log CFU at 2.5 and 5 ppm, respectively. HCl- and lactic acid-produced ClO_2_ reduced *S*. Typhimurium by 4.20 and 5.05 log CFU, and 3.89 and 4.67 log CFU at 2.5 and 5 ppm, respectively. Overall, ClO_2_ solutions generated with malic acid inactivated significantly higher numbers of *S*. Typhimurium than the solutions generated with HCl or lactic acid (*p* < 0.05). 1.74–2.01 and 0.86–1.97 log CFU/cm^2^ *Salmonella* was inactivated in 100 ppm free chlorine and 80 ppm peracetic acid with scrubbing by sponges/microfiber, respectively [35]. Comparing with these results, the microbial reduction on papayas achieved by immersing in ClO_2_ for 5 min seems more effective.

Water treatment only removed 2.56 log CFU of *S*. Typhimurium from papaya surface, whereas 4.47 log CFU of *L. monocytogenes* was removed by water (Figure 2). This may be partially due to that *S*. Typhimurium attached stronger to papaya surfaces than *L. monocytogenes*. In a study conducted by Collignon and Korsten [42], *S*. Typhimurium adhered to peach immediately after contact, whereas *L. monocytogenes* required 60 s for the adhesion. Higher numbers of *S*. Typhimurium cells were observed in one hour than *L. monocytogenes* on peach.

ClO_2_ produced with HCl did not show higher effectiveness in reducing *L. monocytogenes* than water (Figure 2B). ClO_2_ produced using lactic acid had increased bacterial reductions than HCl-produced ClO_2_ at 5 and 10 ppm but with large variations. Malic acid-produced ClO_2_ showed the highest *L. monocytogenes* reduction among all ClO_2_ treatments. However, there was no significant difference between the three tested concentrations. The group treated with ClO_2_ made with malic acid showed statistically higher bacterial reduction than the group treated with ClO_2_ made with HCl (*p* < 0.05). 2.5, 5 and 10 ppm of malic acid-generated ClO_2_ reduced *L. monocytogenes* by 7.20, 6.63 and 8.04 log CFU, respectively. These reductions were higher than the *L. monocytogenes* reductions on apples, lettuce, strawberries and cantaloupe treated with 5 ppm ClO_2_ made with phosphoric acid (~5.6 log CFU) [59]. *L. monocytogenes*-contaminated papayas treated with 200 ppm bleach also showed a relatively large variation with an average reduction of 5.5 log CFU, which was lower than all samples treated with malic acid-generated ClO_2_. However, the concentration of bleach was much higher than that of ClO_2_, indicating the high antimicrobial efficiency of ClO_2_. This result agrees with the higher reduction of *L. monocytogenes* on blueberries treated with 10 ppm ClO_2_ (1.7 log CFU/g) than those treated with 200 ppm chlorine (1.0 log CFU/g) for 5 min [23].

ClO_2_ generated with malic acid inactivated significantly more *S*. Typhimurium and *L. monocytogenes* than ClO_2_ generated with HCl. This result is consistent with our previous observation of the high antimicrobial efficiency of ClO_2_ generated with organic acids. In particular, malic acid-generated ClO_2_ had higher efficacy in killing *S*. Typhimurium and *L. monocytogenes* than HCl-, sodium bisulfate-, citric acid- and lactic acid- generated ClO_2_ [38]. This conclusion was drawn from experiments conducted on bacteria cell suspensions and Romaine lettuce. We hypothesized that synergistic effects between ClO_2_ and the excessive organic acids in the ClO_2_ solutions may contribute to the high antimicrobial efficiency of organic acid-generated ClO_2_. We treated contaminated papayas with individual acid solutions to confirm this hypothesis. Since the pH of ClO_2_ decreased with the increase of its concentration (data not shown), pH values corresponding to 10 ppm ClO_2_ were selected for the decontamination experiments with acids alone. This means the pH of HCl, lactic acid and malic acid solutions were adjusted to 3.15, 3.42 and 3.36, respectively. *S.* Typhimurium on papayas treated with the acids was reduced by 2.45–3.01 log CFU, which was not significantly different from the samples treated with water (Table 4, *p* > 0.05). Similarly, *L. monocytogenes* on papayas treated with the acids was reduced by 3.58–3.91 log CFU and was not significantly different from the samples treated with water (*p* > 0.05). Hence these results confirmed the high antimicrobial effect of ClO_2_ solutions made with malic acid and lactic acid was contributed little by the excessive organic acids, but rather a synergistic effect between ClO_2_ and organic acids. The combination treatment of 2.0% lactic acid and 10 ppm ClO_2_ resulted in higher reductions of *S.* Typhimurium and *L. monocytogenes* on blueberries than the treatments by each sanitizer alone [60]. On papaya, ClO_2_ produced with lactic acid interestingly had similar killing effects to ClO_2_ produced with HCl, yet ClO_2_ produced with malic acid still performed better than that with HCl. In many studies, lactic acid was either better or as good as malic acid in the inactivation of pathogens when used alone as the sanitizers [61,62]. The synergistic effect somehow altered the antimicrobial efficiency of lactic acid and malic acid. Another factor may contribute to the altered antimicrobial efficacy of the organic-acid-generated ClO_2_ compared with HCl-generated ClO_2_ is the intermediate compounds produced in the ClO_2_ solutions. ClO_2_ solution is a mixture of pure ClO_2_ and oxidative chlorine compounds such as ClO^2−^, ClO^3−^, free chlorine (Cl_2_), hypochlorous acid (HOCl) and hypochlorite ion (OCl^−^) [32]. These oxy-species varies in oxidation capacity and stability. Since the *pKa* values of lactic acid and malic acid are different, ClO_2_ solutions generated with the two organic acids reach equilibrium differently and have different intermediate compound compositions. Measures of the intermediate compound compositions and their chemical oxygen demand would help further understand the mechanisms underlining the different antimicrobial efficacies between various ClO_2_ solutions.

Additionally, CFR Sec. 173.300 specifies that ClO_2_ can be used in fresh produce wash with a rinse procedure, and ClO_2_ residue in the wash water of the applied fresh produce should not exceed 3 ppm [25]. EPA also specifies that ClO_2_ is allowed to rinse fruits and vegetables at a concentration of 5 ppm [63]. Some literature also suggests that the residue on the washed produce should not exceed 3 ppm [64,65]. In this study, the ClO_2_ residue on papayas after being treated with 5, 10 and 20 ppm ClO_2_ solutions ranged from 8.0 × 10^−5^ to 6.2 × 10^−3^ mg/kg, which were far below 3 ppm (Table 5). These numbers were also far below the EPA regulation of 0.8 mg/L ClO_2_ residue in public drinking water [27]. Tomatoes and strawberries treated with 0.5 ppm gaseous ClO_2_ for 10 min had 0.09 and 0.37 mg/kg ClO_2_ residue [29]. ClO_2_ residue on produce treated with gaseous ClO_2_ was much higher than ClO_2_ residue on papayas treated with aqueous ClO_2_, providing insights into safety concerns in the application of ClO_2_. However, future studies of ClO^2−^ reside on food matrix treated with ClO_2_ should be carried out as ClO^2−^ and ClO^3−^ are harmful disinfection by-products (DPBs) that can cause anemia and thyroid dysfunction in animals [26].

## 4. Conclusions

To provide potential solutions to the emerging issue of foodborne illness outbreaks associated with whole papayas, this study investigated the survival of *S.* Typhimurium and *L. monocytogenes* on whole papaya during storage at 21 and 7 °C and determined the efficiency of aqueous ClO_2_ in inactivating the two pathogenic bacteria on whole papaya. Temperature played a significant role in the survival and growth of the bacteria on the fruit. *S.* Typhimurium grew by 1.88 log CFU on whole papaya in 14 days at 21 °C and remained at the initial inoculation level at 7 °C. *L. monocytogenes* grew by 0.93 log CFU on papaya during the 1st day of storage at 21 °C; the level remained stable thereafter at 21 °C and at 7 °C. The acid used to produce aqueous ClO_2_ influenced the killing efficiency of ClO_2_ against these pathogenic bacteria. ClO_2_ solutions generated with malic acid reduced significantly higher levels of *S.* Typhimurium and *L. monocytogenes* than the solution generated with HCl. Methodology wise, we optimized the methods for recovering pathogenic bacteria cells from papaya surface, which was a crucial step evaluating bacterial behavior on fresh produce. This study also provided information on ClO_2_ residue on the washed papayas. These results give insights on risk assessment and management of microbiological safety issues associated with whole papaya. Further studies including the intermediate compound compositions in various ClO_2_ solutions and the residue of DPBs on ClO_2_ treated food matrix are suggested to better understand the antimicrobial mechanisms and safety concerns regarding using aqueous ClO_2_.

## Figures and Tables

**Table 1:** *S.* Typhimurium population (log CFU) recovered from papaya surface as affected by homogenization buffer, time (min), speed (rpm) and enumeration methods *.

Buffer	1 Min				5 Min				Average
	150 Rpm		250 Rpm		150 Rpm		250 Rpm		
	XLD	PCA + XLD	XLD	PCA + XLD	XLD	PCA + XLD	XLD	PCA + XLD	
PBS	5.31 ± 0.65	5.34 ± 0.84	4.91 ± 0.38	4.95 ± 0.44	5.14 ± 0.29	5.38 ± 0.18	4.86 ± 0.82	4.81 ± 0.99	5.11 ± 0.57 ^a,b^
PEPT	4.77 ± 0.50	4.80 ± 0.43	4.99 ± 0.52	5.05 ± 0.46	4.57 ± 0.47	4.85 ± 0.67	4.74 ± 0.32	4.54 ± 0.22	4.77 ± 0.40 ^b^
PBS + T	5.08 ± 0.26	5.18 ± 0.30	5.43 ± 0.38	5.55 ± 0.34	5.64 ± 0.46	5.39 ± 0.49	5.29 ± 0.10	5.31 ± 0.07	5.36 ± 0.33 ^a^
PEPT + T	5.08 ± 0.87	5.25 ± 0.74	5.26 ± 0.47	5.45 ± 0.41	5.07 ± 0.34	5.49 ± 0.28	5.39 ± 0.50	5.41 ± 0.24	5.28 ± 0.45 ^a^

* “PBS”, “PEPT”, “PBS + T” and “PEPT + T” stand for phosphate buffered saline, 0.1% peptone water, PBS with 0.2% Tween 80 and 0.1% peptone water with 0.2% Tween 80, respectively. Enumeration methods “XLD” and “PCA + XLD” stand for xylose lysine deoxycholate agar and plate count agar overlaid with XLD, respectively. Numbers are mean ± standard deviation (*n* = 3). No significant interactions were found between the factors. Means in the same column with different superscripts are significantly different (*p* < 0.05).

**Table 2:** *L. monocytogenes* population (log CFU) recovered from papaya surface as affected by homogenization buffer, time (min), speed (rpm) and enumeration methods *.

Buffer	1 Min				5 Min				Average
	150 Rpm		250 Rpm		150 Rpm		250 Rpm		
	MOX	PCA + MOX	MOX	PCA + MOX	MOX	PCA + MOX	MOX	PCA + MOX	
PBS	4.23 ± 0.49	4.49 ± 0.75	4.79 ± 0.17	4.76 ± 0.20	4.60 ± 0.81	4.50 ± 0.74	4.36 ± 0.58	4.35 ± 0.59	4.51 ± 0.52 ^b^
PEPT	5.02 ± 0.21	4.55 ± 0.62	4.84 ± 0.55	4.67 ± 0.45	4.89 ± 0.76	4.30 ± 0.37	4.57 ± 0.60	4.61 ± 0.45	4.68 ± 0.49 ^a,b^
PBS + T	4.97 ± 0.29	4.61 ± 0.33	4.97 ± 0.13	4.93 ± 0.15	4.92 ± 0.48	5.09 ± 0.19	5.08 ± 0.3	4.94 ± 0.01	4.94 ± 0.25 ^a^
PEPT + T	4.96 ± 0.63	4.54 ± 1.03	4.85 ± 0.58	4.88 ± 0.55	4.62 ± 0.83	4.38 ± 0.89	4.55 ± 1.04	4.49 ± 0.97	4.66 ± 0.73 ^a,b^

* “PBS”, “PEPT”, “PBS + T” and “PEPT + T” stand for phosphate buffered saline, 0.1% peptone water, PBS with 0.2% Tween 80 and 0.1% peptone water with 0.2% Tween 80, respectively. Enumeration methods “MOX” and “PCA + MOX” stand for modified Oxford agar and plate count agar overlaid with MOX, respectively. Numbers are mean ± standard deviation (*n* = 3). No significant interactions were found between the factors. Means in the same column with different superscripts are significantly different (*p* < 0.05).

**Table 3:** pH of papaya skin homogenate as affected by homogenization buffer type, time (min) and speed (rpm) *.

Buffer	1 Min		5 Min		Average
	150 Rpm	250 Rpm	150 Rpm	250 Rpm	
PBS	7.19 ± 0.08	7.20 ± 0.09	7.21 ± 0.06	7.22 ± 0.09	7.21 ± 0.07 ^a^
PEPT	6.32 ± 0.07	6.19 ± 0.28	6.37 ± 0.25	6.18 ± 0.05	6.26 ± 0.18 ^b^
PBS + T	7.11 ± 0.06	7.08 ± 0.06	7.44 ± 0.56	7.12 ± 0.06	7.19 ± 0.29 ^a^
PEPT + T	5.88 ± 0.27	5.87 ± 0.21	5.79 ± 0.06	6.03 ± 0.23	5.89 ± 0.20 ^c^
Water	6.05 ± 0.22	6.03 ± 0.17	6.10 ± 0.14	5.82 ± 0.08	6.00 ± 0.17 ^c^

* “PBS”, “PEPT”, “PBS + T” and “PEPT + T” stand for phosphate buffered saline, 0.1% peptone water, PBS with 0.2% Tween 80 and 0.1% peptone water with 0.2% Tween 80, respectively. Numbers are mean ± standard deviation (*n* = 3). No significant interactions were found between the factors. Means in the same column with different superscripts are significantly different (*p* < 0.05).

**Table 4:** *S.* Typhimurium and *L. monocytogenes* reduction (log CFU) by water, HCl, lactic acid and malic acid on whole papayas *.

Acid	*S.* Typhimurium	*L. monocytogenes*
Water	2.41 ± 0.24	3.86 ± 0.09
HCl	3.01 ± 0.42	3.58 ± 0.19
Lactic acid	2.77 ± 0.18	3.64 ± 0.43
Malic acid	2.45 ± 0.15	3.91 ± 0.43

* Numbers are mean ± standard deviation (*n* = 3). No statistical significance was found between treatments within each column.

**Table 5:** ClO_2_ residue (mg/kg) on papaya surface after being washed with ClO_2_ *.

Acid Used to Generate ClO_2_	Concentration of ClO_2_ Wash Solution		
	5 ppm	10 ppm	20 ppm
HCl	7.8×$10{-}^{4}\;\pm \;1.4\times$10^−3^	<3.7×10^−7^	<4.0×10^−7^
Lactic acid	<3.6×10^−7^	8.0×$10{-}^{5}\;\pm \;1.4\times$10^−4^	6.2×$10{-}^{3}\;\pm \;3.9\times$10^−3^
Malic acid	<3.6×10^−7^	<3.3×10^−7^	<3.6×10^−7^

* Numbers are mean ± standard deviation (*n* = 3).

## References

[1] Evans E.A., Ballen F.H., Crane J.H. (2012). An Overview of US Papaya Production, Trade, and Consumption.

[2] (2019). FAOSTAT. http://www.fao.org/faostat/en/#data/QC/visualize.

[3] Gibbs R., Pingault N., Mazzucchelli T., O’Reilly L., MacKenzie B., Green J., Mogyorosy R., Stafford R., Bell R., Hiley L. (2009). An Outbreak of *Salmonella* enterica Serotype Litchfield Infection in Australia Linked to Consumption of Contaminated Papaya. J. Food Prot..

[4] Hassan R., Whitney B., Williams D.L., Holloman K., Grady D., Thomas D., Omoregie E., Lamba K., Leeper M., Gieraltowski L. (2019). Multistate outbreaks of *Salmonella* infections linked to imported Maradol papayas—United States, December 2016–September 2017. Epidemiol. Infect..

[5] FDA (2019). Letter to Papaya Grower, Harvesters, Packers, Distributors, Exporters, Importers, and Retailers Concerning Food-Borne Illness Outbreaks Tied to Papayas. https://www.fda.gov/media/130271/download.

[6] de Oliveira J.G., Vitória A.P. (2011). Papaya: Nutritional and pharmacological characterization, and quality loss due to physiological disorders. An overview. Food Res. Int..

[7] Strawn L.K., Schneider K.R., Danyluk M.D. (2011). Microbial Safety of Tropical Fruits. Crit. Rev. Food Sci. Nutr..

[8] Marik C.M., Zuchel J., Schaffner D.W., Strawn L.K. (2019). Growth and Survival of *Listeria monocytogenes* on Intact Fruit and Vegetable Surfaces During Postharvest Handling: A Systematic Literature Review. J. Food Prot..

[9] CDC (2012). Multistate Outbreak of Listeriosis Linked to Whole Cantaloupes from Jensen Farms. Colorado|Listeria|CDC. https://www.cdc.gov/listeria/outbreaks/cantaloupes-jensen-farms/index.html.

[10] CDC (2015). Multistate Outbreak of Listeriosis Linked to Commercially Produced, Prepackaged Caramel Apples Made from Bidart Bros. Apples|Listeria|CDC. https://www.cdc.gov/listeria/outbreaks/caramel-apples-12-14/index.html.

[11] Poimenidou S.V., Chatzithoma D.-N., Nychas G.-J., Skandamis P.N. (2016). Adaptive Response of *Listeria monocytogenes* to Heat, Salinity and Low pH, after Habituation on Cherry Tomatoes and Lettuce Leaves. PLoS ONE.

[12] Rangel-Vargas E., Luna-Rojo A.M., Cadena-Ramírez A., Torres-Vitela R., Gomez-Aldapa C.A., Villarruel-López A., Téllez-Jurado A., Villagómez-Ibarra J.R., Reynoso-Camacho R., Castro-Rosas J. (2018). Behavior of 11 Foodborne Bacteria on Whole and Cut Mangoes var. Ataulfo and Kent and Antibacterial Activities of Hibiscus sabdariffa Extracts and Chemical Sanitizers Directly onto Mangoes Contaminated with Foodborne Bacteria. J. Food Prot..

[13] Sheng L., Edwards K., Tsai H.-C., Hanrahan I., Zhu M.-J. (2017). Fate of *Listeria monocytogenes* on Fresh Apples under Different Storage Temperatures. Front. Microbiol..

[14] Yuan J., Wang L. (2018). Survival of *Escherichia coli* O157:H7, *Salmonella* spp., and *Listeria monocytogenes* on Fresh and Sliced Green and Golden Kiwifruits. Foodborne Pathog. Dis..

[15] Feng K., Hu W., Jiang A., Xu Y., Sarengaowa, Li X., Bai X. (2015). Growth Potential of *Listeria Monocytogenes* and *Staphylococcus Aureus* on Fresh-Cut Tropical Fruits. J. Food Sci..

[16] Penteado A.L., Leitão M.F. (2004). Growth of *Listeria monocytogenes* in melon, watermelon and papaya pulps. Int. J. Food Microbiol..

[17] Penteado A.L., Leitão M.F. (2004). Growth of *Salmonella* Enteritidis in melon, watermelon and papaya pulp stored at different times and temperatures. Food Control.

[18] Strawn L.K., Danyluk M.D. (2010). Fate of *Escherichia coli* O157:H7 and *Salmonella* spp. on fresh and frozen cut mangoes and papayas. Int. J. Food Microbiol..

[19] Singh A., Yemmireddy V. (2021). Fate of *Salmonella* spp. in fresh-cut papaya (*Carica papaya* L.) at different storage temperature and relative humidity. LWT.

[20] Huff K., Boyer R., Denbow C., O’Keefe S., Williams R. (2012). Effect of Storage Temperature on Survival and Growth of Foodborne Pathogens on Whole, Damaged, and Internally Inoculated Jalapeños (*Capsicum annuum* var. *annuum*). J. Food Prot..

[21] Penteado A.L., Eblen B.S., Miller A.J. (2004). Evidence of *Salmonella* Internalization into Fresh Mangos during Simulated Postharvest Insect Disinfestation Procedures. J. Food Prot..

[22] Perez-Rodriguez F., Begum M., Johannessen G. (2014). Study of the cross-contamination and survival of *Salmonella* in fresh apples. Int. J. Food Microbiol..

[23] Tadepalli S., Bridges D.F., Driver R., Wu V.C. (2018). Effectiveness of different antimicrobial washes combined with freezing against *Escherichia coli* O157:H7, *Salmonella* Typhimurium, and *Listeria monocytogenes* inoculated on blueberries. Food Microbiol..

[24] U.S. Food and Drug Administration (2020). CFR173.300. CFR—Code of Federal Regulations Title 21. https://www.accessdata.fda.gov/scripts/cdrh/cfdocs/cfcfr/cfrsearch.cfm?fr=173.300.

[25] Gómez-López V.M., Rajkovic A., Ragaert P., Smigic N., Devlieghere F. (2009). Chlorine dioxide for minimally processed produce preservation: A review. Trends Food Sci. Technol..

[26] Van Haute S., Tryland I., Escudero C., Vanneste M., Sampers I. (2017). Chlorine dioxide as water disinfectant during fresh-cut iceberg lettuce washing: Disinfectant demand, disinfection efficiency, and chlorite formation. LWT.

[27] U.S. Environmental Protection Agency National Primary Drinking Water Regulations. https://www.epa.gov/ground-water-and-drinking-water/national-primary-drinking-water-regulations#one.

[28] Adhikari A., Chhetri V., Bhattacharya D., Cason C., Luu P., Suazo A. (2019). Effectiveness of daily rinsing of alfalfa sprouts with aqueous chlorine dioxide and ozonated water on the growth of *Listeria monocytogenes* during sprouting. Lett. Appl. Microbiol..

[29] Banach J., van Overbeek L., Groot M.N., van der Zouwen P., van der Fels-Klerx H. (2018). Efficacy of chlorine dioxide on *Escherichia coli* inactivation during pilot-scale fresh-cut lettuce processing. Int. J. Food Microbiol..

[30] Ni Tan J., Hwang C.-A., Huang L., Wu V.C.H., Hsiao H.-I. (2020). In Situ Generation of Chlorine Dioxide for Decontamination of *Salmonella, Listeria monocytogenes*, and Pathogenic *Escherichia coli* on Cantaloupes, Mung Beans, and Alfalfa Seeds. J. Food Prot..

[31] Chen Z., Zhu C., Han Z. (2011). Effects of aqueous chlorine dioxide treatment on nutritional components and shelf-life of mulberry fruit (*Morus alba* L.). J. Biosci. Bioeng..

[32] Trinetta V., Vaidya N., Linton R., Morgan M. (2010). Evaluation of Chlorine Dioxide Gas Residues on Selected Food Produce. J. Food Sci..

[33] Boonyaritthongchai P., Techavuthiporn C., Cumsingnok T. (2018). Effect of Acidified Sodium Chlorite and Packaging on Microbial Reduction and Quality Maintenance of Shredded Green Papaya.

[34] Yeoh W.K., Ali A., Forney C. (2014). Effects of ozone on major antioxidants and microbial populations of fresh-cut papaya. Postharvest Biol. Technol..

[35] Gu G., Bolten S., Mendes-Oliveira G., Zhou B., Teng Z., Pearlstein D., Luo Y., Millner P., Nou X. (2020). *Salmonella* inactivation and sponge/microfiber mediated cross-contamination during papaya wash with chlorine or peracetic acid as sanitizer. Food Microbiol..

[36] Gordon G., Rosenblatt A.A. (2005). Chlorine Dioxide: The Current State of the Art. Ozone Sci. Eng..

[37] Kim H., Kang Y., Beuchat L.R., Ryu J.-H. (2008). Production and stability of chlorine dioxide in organic acid solutions as affected by pH, type of acid, and concentration of sodium chlorite, and its effectiveness in inactivating Bacillus cereus spores. Food Microbiol..

[38] Dong L., Li Y. Comparison of aqueous chlorine dioxide generated with different acids on reducing foodborne pathogenic bacteria. Proceedings of the International Association for Food Protection 2020 Annual Meeting.

[39] Lang M.M., Harris L.J., Beuchat L.R. (2004). Evaluation of Inoculation Method and Inoculum Drying Time for Their Effects on Survival and Efficiency of Recovery of *Escherichia coli* O157:H7, *Salmonella*, and *Listeria monocytogenes* Inoculated on the Surface of Tomatoes. J. Food Prot..

[40] Kim S.-R., Yoon Y., Kim W.-I., Park K.-H., Yun H.-J., Chung D.H., Yun J.C., Ryu K.Y. (2012). Comparison of Sample Preparation Methods for the Recovery of Foodborne Pathogens from Fresh Produce. J. Food Prot..

[41] Tripathi S., Suzuki J.Y., Carr J.B., McQuate G.T., Ferreira S.A., Manshardt R.M., Pitz K.Y., Wall M.M., Gonsalves D. (2011). Nutritional composition of Rainbow papaya, the first commercialized transgenic fruit crop. J. Food Compos. Anal..

[42] Collignon S., Korsten L. (2010). Attachment and Colonization by *Escherichia coli* O157:H7, *Listeria monocytogenes*, *Salmonella* enterica subsp. *enterica* serovar Typhimurium, and *Staphylococcus aureus* on Stone Fruit Surfaces and Survival through a Simulated Commercial Export Chain. J. Food Prot..

[43] Wu V.C.H. (2008). A review of microbial injury and recovery methods in food. Food Microbiol..

[44] Zhou L., Paull R.E., Chen N.J. (2014). Papaya: Postharvest Quality-Maintenance Guidelines. Fruit, Nut, and Beverage Crops.

[45] Visvalingam J., Holley R.A. (2018). Evaluation of chlorine dioxide, acidified sodium chlorite and peroxyacetic acid for control of *Escherichia coli* O157:H7 in beef patties from treated beef trim. Food Res. Int..

[46] Wu V.C., Rioux A. (2010). A simple instrument-free gaseous chlorine dioxide method for microbial decontamination of potatoes during storage. Food Microbiol..

[47] Brandl M.T., Huynh S. (2014). Effect of the Surfactant Tween 80 on the Detachment and Dispersal of *Salmonella* enterica Serovar Thompson Single Cells and Aggregates from Cilantro Leaves as Revealed by Image Analysis. Appl. Environ. Microbiol..

[48] Tian X., Yu Q., Shao L., Li X., Dai R. (2018). Sublethal injury and recovery of *Escherichia coli* O157:H7 after ohmic heating. Food Control.

[49] Ma Q., Zhang Y., Critzer F., Davidson P.M., Zhong Q. (2016). Quality attributes and microbial survival on whole cantaloupes with antimicrobial coatings containing chitosan, lauric arginate, cinnamon oil and ethylenediaminetetraacetic acid. Int. J. Food Microbiol..

[50] Koseki S., Nakamura N., Shiina T. (2015). Comparison of Desiccation Tolerance among *Listeria monocytogenes*, *Escherichia coli* O157:H7, *Salmonella* enterica, and *Cronobacter sakazakii* in Powdered Infant Formula. J. Food Prot..

[51] Dhowlaghar N., Tang J., Zhu M.-J. (2021). Thermal inactivation of *Salmonella*, *Listeria monocytogenes* and *Enterococcus faecium* NRRL B-2354 in desiccated shredded coconut. LWT.

[52] Mathew E.N., Muyyarikkandy M.S., Kuttappan D., Amalaradjou M.A. (2018). Attachment of *Salmonella* enterica on Mangoes and Survival Under Conditions Simulating Commercial Mango Packing House and Importer Facility. Front. Microbiol..

[53] Bardsley C.A., Truitt L.N., Pfuntner R.C., Danyluk M.D., Rideout S.L., Strawn L.K. (2019). Growth and Survival of *Listeria monocytogenes* and *Salmonella* on Whole and Sliced Cucumbers. J. Food Prot..

[54] Behrsing J., Jaeger J., Horlock F., Kita N., Franz P., Premier R. (2003). Survival of *Listeria innocua*, *Salmonella* salford and *Escherichia coli* on the surface of fruit with inedible skins. Postharvest Biol. Technol..

[55] Castro-Rosas J., Gomez-Aldapa C., Acevedo-Sandoval O.A., Ramírez C.A.G., Villagomez-Ibarra J.R., Hernández N.C., Villarruel-Lopez A., Torres-Vitela M.D.R. (2011). Frequency and Behavior of *Salmonella* and *Escherichia coli* on Whole and Sliced Jalapeño and Serrano Peppers. J. Food Prot..

[56] Knudsen D.M., Yamamoto S.A., Harris L.J. (2001). Survival of *Salmonella* spp. and *Escherichia coli* O157:H7 on Fresh and Frozen Strawberries. J. Food Prot..

[57] Salazar J.K., Carstens C.K., Bathija V.M., Narula S.S., Parish M., Tortorello M.L. (2016). Fate of *Listeria monocytogenes* in Fresh Apples and Caramel Apples. J. Food Prot..

[58] Chan Y.C., Wiedmann M. (2008). Physiology and Genetics of *Listeria Monocytogenes* Survival and Growth at Cold Temperatures. Crit. Rev. Food Sci. Nutr..

[59] Rodgers S.L., Cash J.N., Siddiq M., Ryser E.T. (2004). A Comparison of Different Chemical Sanitizers for Inactivating *Escherichia coli* O157:H7 and *Listeria monocytogenes* in Solution and on Apples, Lettuce, Strawberries, and Cantaloupe. J. Food Prot..

[60] Tadepalli S., Bridges D.F., Anderson R., Zhang R., Wu V.C. (2019). Synergistic effect of sequential wash treatment with two different low-dosage antimicrobial washes in combination with frozen storage increases *Salmonella* Typhimurium and *Listeria monocytogenes* reduction on wild blueberries. Food Control.

[61] Almasoud A., Hettiarachchy N., Rayaprolu S., Babu D., Kwon Y.M., Mauromoustakos A. (2016). Inhibitory effects of lactic and malic organic acids on autoinducer type 2 (AI-2) quorum sensing of *Escherichia coli* O157:H7 and *Salmonella* Typhimurium. LWT.

[62] Mohan A., Pohlman F. (2016). Role of organic acids and peroxyacetic acid as antimicrobial intervention for controlling *Escherichia coli* O157:H7 on beef trimmings. LWT.

[63] EPA (2006). Reregistration Eligibility Decision (RED) for Chlorine Dioxide and Sodium Chlorite (Case 4023). https://www3.epa.gov/pesticides/chem_search/reg_actions/reregistration/red_PC-020503_3-Aug-06.pdf.

[64] Mathew E.N., Muyyarikkandy M.S., Bedell C., Amalaradjou M.A. (2018). Efficacy of Chlorine, Chlorine Dioxide, and Peroxyacetic Acid in Reducing *Salmonella* Contamination in Wash Water and on Mangoes Under Simulated Mango Packinghouse Washing Operations. Front. Sustain. Food Syst..

[65] Pao S., Kelsey D.F., Long W. (2009). Spray washing of tomatoes with chlorine dioxide to minimize *Salmonella* on inocu-lated fruit surfaces and cross-contamination from revolving brushes†. J. Food Prot..

